# Effect of Malaria on Blood Levels of Vitamin E: A Systematic Review and Meta-Analysis

**DOI:** 10.3390/nu15153472

**Published:** 2023-08-05

**Authors:** Manas Kotepui, Frederick Ramirez Masangkay, Aongart Mahittikorn, Kwuntida Uthaisar Kotepui

**Affiliations:** 1 Medical Technology, School of Allied Health Sciences, Walailak University, Tha Sala, Nakhon Si Thammarat 10400, Thailand; manas.ko@wu.ac.th; 2 Department of Medical Technology, Faculty of Pharmacy, University of Santo Tomas, Manila 1008, Philippines; 3 Department of Protozoology, Faculty of Tropical Medicine, Mahidol University, Bangkok 10400, Thailand

**Keywords:** malaria, *Plasmodium*, antioxidant, vitamin E, tocopherol, systematic review, meta-analysis

## Abstract

Vitamin E has an antioxidant property and is associated with protection against malaria. The current study used systematic review and meta-analysis approaches examining the variance in blood levels of vitamin E in malaria patients as compared with uninfected individuals. The protocol for the systematic review was registered with PROSPERO (CRD4202341481). Searches for pertinent studies were carried out on Embase, MEDLINE, Ovid, PubMed, Scopus, ProQuest, and Google Scholar. The combined effect estimate (Cohen’s d) of the difference in vitamin E levels in malaria patients as compared with uninfected individuals was estimated using the random effects model. The searches yielded 2009 records, and 23 studies were included in the systematic review. The majority of the studies (80%) found that vitamin E levels were significantly lower in malaria patients than those who were not infected. Overall, the results revealed a significant reduction in blood levels of vitamin E in malaria patients when compared with uninfected individuals (*p* < 0.01, Cohen’s d: −2.74, 95% CI: −3.72–(−1.76), I^2^: 98.69%, 21 studies). There was a significant reduction in blood levels of vitamin E in patients suffering from severe malaria, in comparison with those experiencing less severe forms of the disease (*p* < 0.01, Cohen’s d: −0.56, 95% CI: −0.85–(−0.26), I^2^: 0%, 2 studies), but no variation in blood levels of vitamin E among patients suffering from either *P. falciparum* or *P. vivax* malaria (*p* = 0.13, Cohen’s d: −1.15, 95% CI: −2.62–0.33, I^2^: 93.22%, 3 studies). In summary, the present study strongly suggests that vitamin E levels are significantly reduced in malaria patients, with a more pronounced decrease observed in cases of severe malaria. However, the type of malaria parasite, specifically *P. falciparum* or *P. vivax*, did not appear to influence the levels of vitamin E. This study highlights the potential role of vitamin E in the pathogenesis of malaria and suggests that improved vitamin E status might be beneficial for improving disease outcomes.

## 1. Introduction

Malaria is a protozoan disease caused by infection with *Plasmodium vivax, Plasmodium falciparum*, *Plasmodium ovale*, *Plasmodium malariae*, or *Plasmodium knowlesi* through the bite of Anopheline mosquitoes [1,2,3]. Despite continuing efforts in malaria prevention and control, the disease affects more than 300 million to 500 million people worldwide, particularly children in Africa. In the year 2021, malaria cases worldwide reached 247 million, with an estimated 619,000 malaria deaths [3]. *Plasmodium* infection leads to the production of free radicals through oxidative stress, which is one of the pathogenesis mechanisms caused by *Plasmodium* parasites [4,5]. Although the role of oxidative stress in relation to malaria remains somewhat elusive, studies have reported an association between the production of reactive oxygen species (ROS) and the progression to severe malaria [4,6,7]. Therefore, combining antimalarial drugs with antioxidant agents could be a promising approach to enhance the efficacy of malaria case management and control strategies.

Vitamin E, a fat-soluble nutrient, is composed of four tocopherols and four tocotrienols. A well-known function of vitamin E is its antioxidant property, which prevents lipid peroxidation of the cell membrane [8]. In a mouse model, there is evidence to suggest that vitamins E and A may possess antimalarial properties [9]. Specifically, vitamin E, when administered at the dosage and via the route employed, was effective in preventing anemia induced by *P. berghei*, as well as in mitigating alterations in both relative organ weight and antioxidant status in mice [9]. Furthermore, a combination of vitamins C and E has been indicated to have potential benefits in ameliorating *P. berghei*-induced anemia and other pathological effects in mice [10]. On the contrary, a study in a mouse model showed that mice with vitamin E deficiency in combination with chloroquine significantly improved their survival rates when infected with *Plasmodium* [11]. In addition, *P. falciparum* was reported to use vitamin E to avoid oxidative stress and protect itself from the radicals generated by antimalarial drugs [12]. Studies on HIV-infected Tanzanian women showed that multivitamin supplementation can reduce the risk of presumptively diagnosed clinical malaria and reduce high parasitemia [13,14]. Although the relationship between vitamin E and its role in malaria pathogenesis is demonstrated through studies conducted both in vitro and in vivo, the role of vitamin E in human participants is inconsistent, and the conclusion of the relationship was based on studies with a small number of participants. As a result, the current study used systematic review and meta-analysis approaches to investigate the difference in vitamin E levels between patients infected with *Plasmodium* species and those who were not. Furthermore, the difference in vitamin E levels between different clinical malaria and *Plasmodium* species was also investigated. The outcome of this study will offer necessary insights to direct the potential benefit of vitamin E as a vitamin supplementation to patients with malaria in order to manage the disease’s poor outcomes or to participants who reside in endemic areas in order to help prevent *Plasmodium* infection.

## 2. Methods

### 2.1. Registration, Guidelines for Reporting, and Review Questions

The protocol for the systematic review was registered with PROSPERO (CRD4202341481). The systematic review and meta-analysis were reported following the PRISMA 2020 statement [15]. The systematic review questions followed the population, intervention, comparator(s), and outcomes (PICO) framework [16]. P was patients with malaria; I was none; C were patients without malaria; and O were vitamin E levels.

### 2.2. Eligibility Criteria

The eligibility criteria include both the inclusion and exclusion criteria. The inclusion criteria were as follows: (i) the study must have reported on vitamin E levels in malaria patients and made a comparison with individuals who were not infected with malaria, (ii) vitamin E levels must have been measured before the patients underwent any treatment for malaria, and (iii) the study could include any form of vitamin E. The exclusion criteria were animal studies; in vitro studies; studies that do not provide data on vitamin E levels in malaria patients; studies for which the full text is not available; studies involving patients who received vitamin supplementation prior to the study; studies focusing exclusively on severe malaria and vitamin E levels; studies focusing on vitamin E levels in conditions other than malaria; studies that include participants that overlap with those in another study; studies in which data on vitamin E levels in malaria patients could not be extracted; studies that measured vitamin E levels after malaria treatment; review articles; and conference abstracts.

### 2.3. Search Strategy

The searches were conducted in six databases including Embase, MEDLINE, Ovid, PubMed, Scopus, and ProQuest. The synonyms of the search terms “Vitamin E” and “malaria” that were used in the search strategy were identified from Medical Subject Headings (MeSH). The identified search terms were combined with Boolean operators such as (“Vitamin E” OR tocopherols OR alpha-tocopherol OR beta-tocopherol OR gamma-tocopherol OR tocotrienols) AND (malaria OR *Plasmodium* OR “Remittent Fever” OR “Marsh Fever” OR paludism). The details of the search strategy in each database are demonstrated in Table S1. The searches were not restricted to the English language or by publication dates of the articles. A search was also executed on Google Scholar to discover pertinent studies not cataloged in the primary databases. The final date for searches in main databases and Google Scholar was 31 March 2023.

### 2.4. Study Selection

All the articles collected from the databases were transferred into EndNote 20.0 software (Clarivate Analytics, Philadelphia, PA, USA). After removing duplicates, studies were screened based on their titles and abstracts for relevance. After irrelevant studies were excluded, the remaining studies’ unabridged texts underwent screening for the final selection based on the eligibility criteria. Studies were excluded for a variety of reasons, including failure to meet the criteria. Two review authors independently conducted the screening and examination of the articles, and any discrepancies in the selection of studies were resolved through discussion.

### 2.5. Data Extraction

The same two review authors extracted the data from selected studies into the standard spreadsheet. Any discrepancies during data extraction were resolved through discussion and consensus between the authors. The following information was collected from every single study: first author’s name, publication year, design of the study, area and continent, year of conduction, study participants (children, adults, pregnant women, or mixed), median/mean age, clinical status (asymptomatic, severe, non-severe, or mixed), vitamin E levels (higher or lower between groups), vitamin E levels (mean and standard deviation, median and interquartile range), parasite density, methods for malaria detection (microscopy, rapid diagnostic test, molecular, or combination), and methods for vitamin E measurement.

### 2.6. Quality Assessment

The quality of the studies was evaluated utilizing the Joanna Briggs Institute (JBI) critical appraisal tools [17]. There were 8 checklists for cross-sectional studies, 10 checklists for case-control studies, and 11 checklists for cohort studies. On the basis of the existence or lack of each item on the checklists, the studies were assigned a rating of yes, no, ambiguous, or not applicable. The assessment was conducted by the same two review authors, and any differences were solved through discussion and agreement.

### 2.7. Data Synthesis

The combined effect estimate (Cohen’s d) with 95% confidence intervals (CIs) of vitamin E levels between malaria patients and those who were not infected, between those with severe malaria and those experiencing less severe forms of the disease, and between patients suffering from *P. falciparum* malaria and those suffering from *P. vivax* malaria, was estimated using the random effects model as previously described [18]. The heterogeneity between studies was evaluated using the Cochran Q and I^2^ statistics [19]. A *p*-value of less than 0.10 for the Cochran Q test demonstrated significant heterogeneity [20]. In addition, levels of heterogeneity were categorized into <25%, 25–75%, and >75%, which indicated low, moderate, and high levels of heterogeneity, respectively [21].

A meta-regression analysis was conducted to assess the potential source of heterogeneity between the meta-analysis’s included studies. Subgroup analyses were carried out to compare the differential effects based on characteristics of studies (publication years, study design, continent, country), participants (*Plasmodium* species, clinical status), the diagnostic test for malaria, and forms of vitamin E. A leave-one-out meta-analysis was employed for the purpose of sensitivity analysis to identify any outlier and measure the statistical validity of the summary meta-analysis [22]. The interpretation of a funnel plot asymmetry [23], Egger’s test [24], and contoured enhanced funnel plot [25] were employed to assess publication bias. The meta-analysis was carried out using Stata 17 and Stata 18 (StataCorp LLC, College Station, TX, USA).

## 3. Results

### 3.1. Search Results

The searches yielded 2009 records from ProQuest (*n* = 889), Ovid (*n* = 338), Embase (*n* = 288), Scopus (*n* = 283), MEDLINE (*n* = 116), and PubMed (*n* = 95). After 1362 nonrelated records were excluded during the title and abstract screening process, 133 records were further examined for their full texts against the eligibility criteria. Among those records, seventeen records met the eligibility criteria. The remaining studies were excluded based on the following criteria: animal studies (*n* = 54), in vitro studies (*n* = 21), reviews (*n* = 15), no data on vitamin E in malaria (*n* = 10), vitamin E in non-malaria (*n* = 4), no full text (*n* = 4), vitamin supplementation (*n* = 3), conference abstracts (*n* = 1), vitamin E in severe malaria only (*n* = 1), studies using overlapping participants (*n* = 1), vitamin E after treatment (*n* = 1), and vitamin E in malaria were unable to be extracted (*n* = 1).

**Figure 1:**
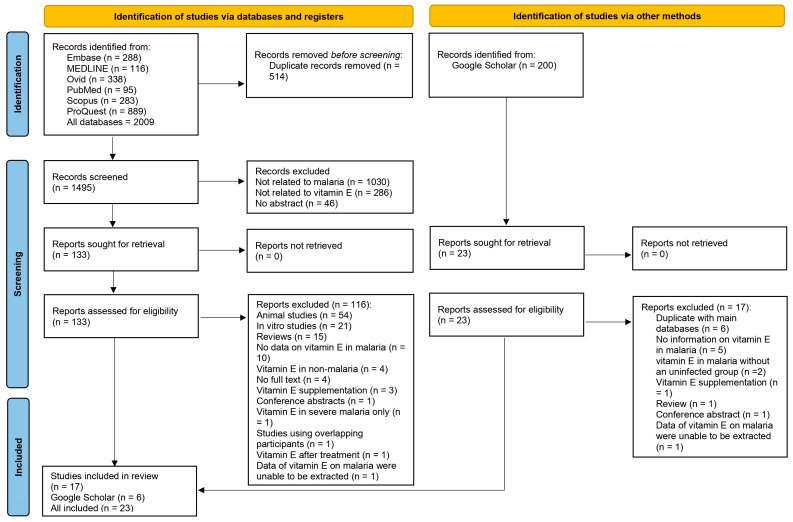
Study flow diagram.

The searches on Google Scholar identified 23 potentially relevant studies, and 6 met the eligibility criteria. The following studies were excluded: duplicates with main databases (*n* = 6), those with no information on vitamin E in malaria (*n* = 5), those on vitamin E in malaria without an uninfected group (*n* = 2), a study for which data were unable to be extracted (*n* = 1), a study on vitamin E supplementation (*n* = 1), a review (*n* = 1), and a conference abstract (*n* = 1). Overall, 23 studies were included in the systematic review and meta-analysis (Figure 1).

### 3.2. Characteristics and Critical Appraisal of the Studies

The characteristics of 23 studies are shown in Table 1. Briefly, most studies were published between 2011 and 2022 (39.1%), followed by 2000–2010 (30.4%), and before 2000 (30.4%). The majority of the studies were case-control (47.8%), followed by cross-sectional studies (43.5%), and cohort studies (8.70%). Most studies were conducted in Africa (56.5%), Asia (34.8%), and Europe (8.70%). Among studies that were conducted in Africa (13 studies) and Asia (8 studies), Nigeria and India accounted for the majority of studies with 92.3% and 62.5%, respectively. The majority of the studies enrolled children (39.1%), followed by adults (30.4%), and pregnant women (17.4%). The majority of the studies included patients infected with *P. falciparum* (69.6%). For clinical symptoms, the majority of the studies enrolled participants with symptomatic malaria (69.6%). The microscopic method was the main method for identifying *Plasmodium* species among the studies (95.7%). Lastly, the majority of studies measured vitamin E in which form of vitamin E was not specified (52.2%), and the remaining studies measured α-tocopherol (39.1%) and total tocopherols (8.70%). S2 illustrates the specifics of each study. The risk of bias of the included studies that were evaluated utilizing the JBI case-control critical appraisal tool, cohort, and correctional studies is demonstrated in Table S3.

### 3.3. Vitamin E Levels in Malaria Patients Compared with Those Who Were Not Infected

Out of 22 studies examining the vitamin E levels in malaria patients compared with those uninfected [26,27,28,29,30,31,32,33,34,35,36,37,38,39,40,41,42,43,44,45,46,47], the majority (18 out of 22 studies, or 81.8%) found that vitamin E levels were significantly lower in malaria patients [26,27,28,29,30,31,32,33,34,35,36,37,38,39,40,41,42,43]. Three studies found no significant difference in vitamin E levels between the two groups [44,45,46], while one study reported increased vitamin E levels in malaria patients compared with those who were not infected [47]. One study investigated vitamin E levels in different levels of parasite density and found that vitamin E levels increased significantly as parasite density increased [48].

**Figure 2:**
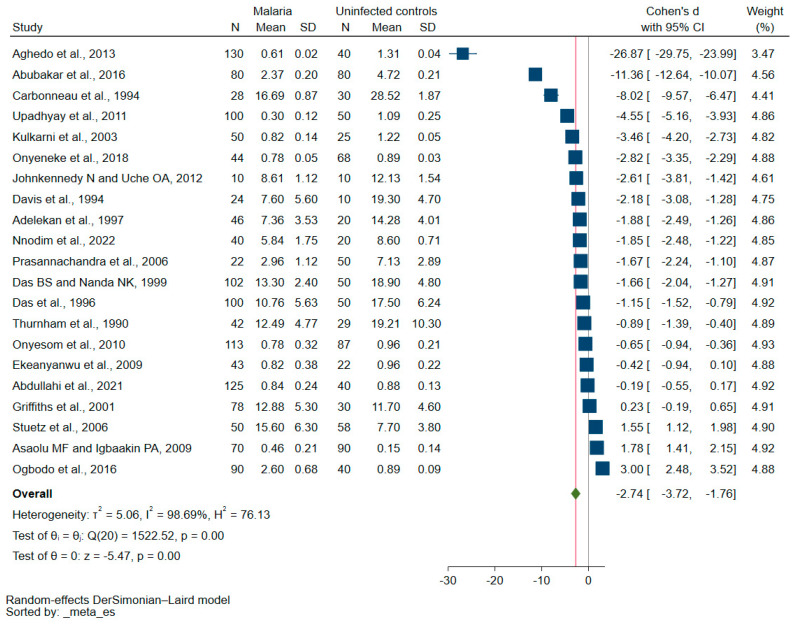
The forest plot illustrates the variation in blood vitamin E levels between malaria patients and uninfected controls. Explanations for symbols: blue square, Cohen’s d for each study; green diamond, pooled Cohen’s d. Abbreviations: CI stands for confidence interval; N represents the number of populations enrolled; SD represents the standard deviation. References: [26,27,28,29,30,31,32,33,35,36,37,38,39,40,41,42,43,44,45,47,48].

The quantitative synthesis was performed using the quantitative data (mean ± SD, median ± IQR) on vitamin E in both malaria patients and those who were not infected from 21 studies [26,27,28,29,30,31,32,33,35,36,37,38,39,40,41,42,43,44,45,47,48]. The meta-analysis showed decreased vitamin E levels in malaria patients when compared with those who were not infected (*p* < 0.01, Cohen’s d: −2.74, 95% CI: −3.72–(−1.76), I^2^: 98.69%, 21 studies, Figure 2).

A meta-regression analysis incorporating variables such as publication year, study design, the continent where the study was conducted, participants’ group, and the species of *Plasmodium*, clinical status, diagnostic method for malaria, and forms of vitamin E showed that continent, participants’ group, *Plasmodium* species, and forms of vitamin E affected the pooled effect estimate (*p* < 0.05) (Table S4). The subgroup analysis indicates that vitamin E levels are generally lower in malaria patients compared with noninfected individuals across different continents, participant groups, and *Plasmodium* species, with a few exceptions. Specifically, decreased vitamin E levels were observed in malaria patients in both Africa and Asia. When considering the participants’ groups, decreased vitamin E levels were noted in studies involving children and adults, but not in studies focusing on pregnant women. For *Plasmodium* species, the trend of decreased vitamin E levels was seen in studies concerning *P. falciparum* as well as those involving both *P. vivax* and *P. falciparum*. However, no significant difference was observed in studies where the *Plasmodium* species was not specified. Finally, when analyzing the forms of vitamin E, a decrease was observed in studies that did not specify the form of vitamin E, while no significant difference was found in studies measuring α-tocopherol or total tocopherols (Table 2). 

### 3.4. Difference in Vitamin E Levels between Patients with Severe Malaria and Those Experiencing Less Severe Forms of the Disease

**Figure 3:**
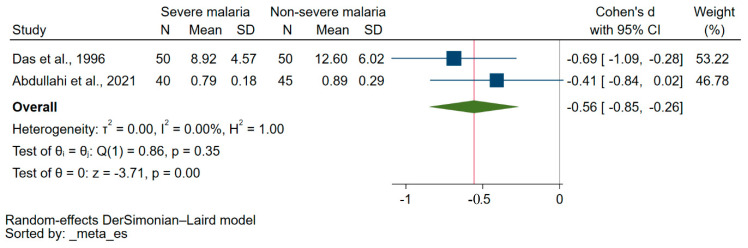
The variation in blood vitamin E levels between patients with severe malaria and those experiencing less severe forms of the disease is illustrated by the forest plot. Explanations for symbols: blue square, Cohen’s d for each study; green diamond, pooled Cohen’s d. Abbreviations: CI stands for confidence interval; N represents the number of patients enrolled; SD represents the standard deviation. References: [32,44].

Two studies reported the quantitative data on vitamin E levels between patients with severe malaria and those experiencing less severe forms of the disease [32,44]. A study showed no difference in the serum vitamin E levels between patients with severe malaria and those experiencing less severe forms of the disease [44]. Another study showed that vitamin E levels were significantly lower in patients with severe malaria than those experiencing less severe forms of the disease [32]. Overall, the results revealed a significant reduction in blood levels of vitamin E levels in patients with severe malaria in comparison with those experiencing less severe forms of the disease (*p* < 0.01, Cohen’s d: −0.56, 95% CI: −0.85–(−0.26), I^2^: 0%, 2 studies, Figure 3).

### 3.5. Difference in Vitamin E Levels among Patients Suffering from either P. falciparum or P. vivax Malaria

**Figure 4:**
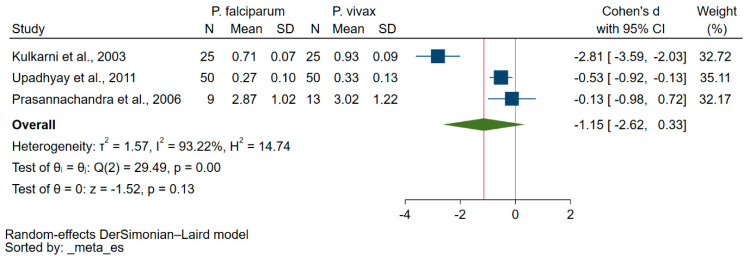
The fluctuation in blood vitamin E levels among patients infected with either *P. falciparum* or *P. vivax* malaria is illustrated by the forest plot. Explanations for symbols: blue square, Cohen’s d for each study; green diamond, pooled Cohen’s d. Abbreviations: CI represents confidence interval; N represents the number of patients enrolled; SD represents the standard deviation. References: [37,41,43].

Three studies reported the quantitative data on vitamin E levels among patients suffering from either *P. falciparum* or *P. vivax* malaria [37,41,43]. Overall, the meta-analysis revealed no substantial variation in blood vitamin E levels in patients infected with either *P. falciparum* or *P. vivax* malaria (*p* = 0.13, Cohen’s d: −1.15, 95% CI: −2.62–0.33, I^2^: 93.22%, 3 studies, Figure 4).

### 3.6. Sensitivity Analysis

**Figure 5:**
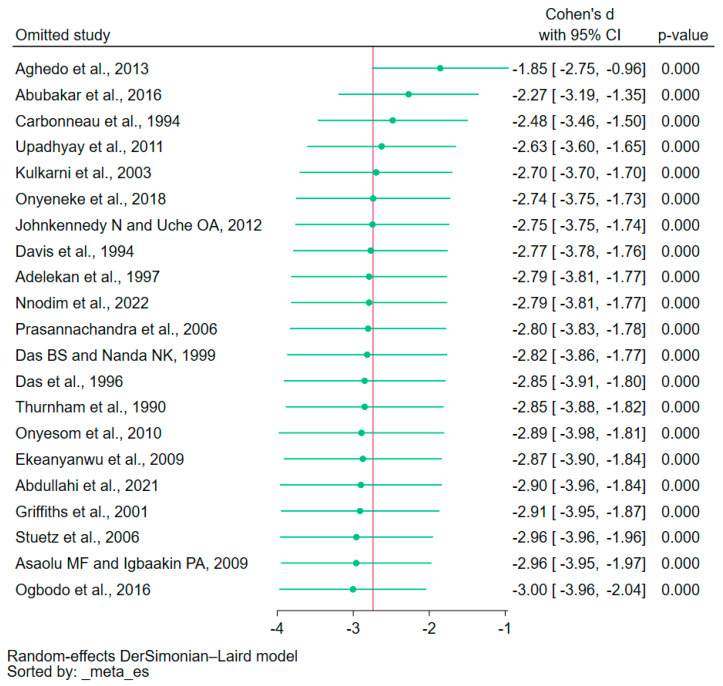
The leave-one-out meta-analysis showing a significant reduction in vitamin E levels in patients with malaria as compared with uninfected controls in each rerun analysis (*p* < 0.05). Explanations for symbols: green circle, pooled Cohen’s d for each re-run analysis; red vertical line, overall Cohen’s d estimated from all re-run analyses. Abbreviation: CI stands for confidence interval. References: [26,27,28,29,30,31,32,33,35,36,37,38,39,40,41,42,43,44,45,47,48].

The leave-one-out meta-analysis indicated that no single study had a significant impact on the pooled effect estimate in the comparison of vitamin E levels between malaria patients and uninfected individuals (*p* < 0.05, Figure 5). This suggests that the meta-analysis results were stable and reliable. The leave-one-out meta-analysis in the meta-analysis of vitamin E levels between patients with severe malaria and those experiencing less severe forms of the disease, and among patients infected with either *P. falciparum* or *P. vivax* malaria, was not possible to be executed owing to the limited quantity of studies incorporated in the meta-analysis.

### 3.7. Publication Bias

**Figure 6:**
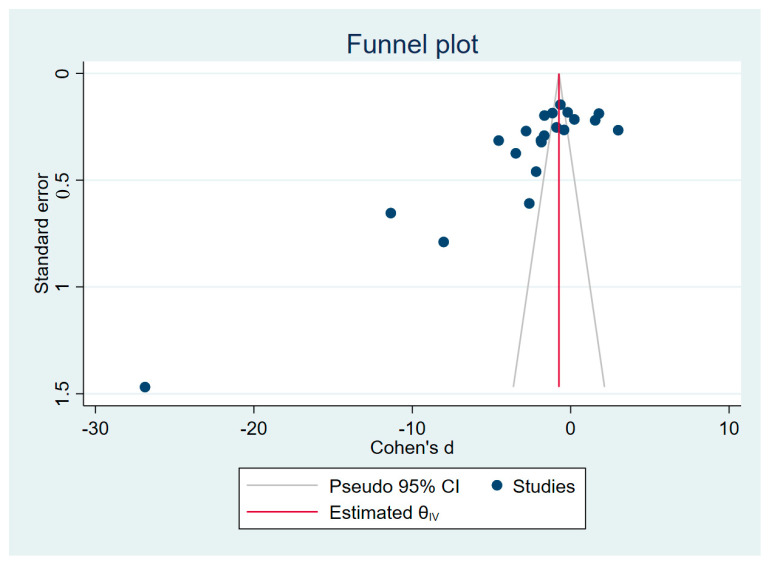
An asymmetrical spread of the effect estimate (SMD) from the middle line (red). The funnel plot was generated using the meta-analysis data from blood vitamin E levels between malaria patients and uninfected individuals. Abbreviation: CI, confidence interval.

There were asymmetrical distributions of effect estimates in the funnel plot of the meta-analysis of vitamin E levels between malaria patients and those who were not infected (Figure 6). Egger’s test outcomes revealed a small-study effect (*p* < 0.01), indicating reporting bias among the studies included in the meta-analysis. The funnel plot’s asymmetry was caused by the effect estimates’ heterogeneity, as seen by the distributions of effect estimates beyond the significant area of the funnel plot (*p* < 0.05, Supplementary Figure S1). Trim and fill analysis demonstrated decreased vitamin E levels in malaria patients as compared with those who were not infected (Cohen’s d: −0.74, 95% CI: −0.85–(−0.63), 30 studies).

## 4. Discussion

The evidence of the systematic review and meta-analysis showed decreased vitamin E levels in malaria patients as compared with the uninfected group. The reduction in vitamin E levels during *Plasmodium* infection may indicate the reaction of the host’s defensive system to the production of reactive oxygen species or their metabolites. The decrease in vitamin E levels in patients with malaria may indicate an increased requirement, increased destruction, or increased utilization of vitamin E during malaria infection [43]. The transfer of vitamin E to the membranes of red blood cells to combat the rise in oxidative stress during the acute phase of the *Plasmodium* infection may be the cause of the drop in vitamin E levels in malaria patients [43]. Furthermore, vitamin E can protect erythrocyte membranes from oxidative stress, as shown by the clinical trial investigating vitamin E supplementation among healthy people [49]. The improvement in erythrocyte membrane fluidity and reduction in erythrocyte hemolysis is another proposed mechanism for protection against *Plasmodium* infection by vitamin E [49]. Vitamin E in combination with other antioxidant systems was consumed to counteract the higher levels of oxidative stress, and its levels diminished, suggesting the mechanisms of the body can combat or neutralize the effect of oxidative stress during *Plasmodium* infection. The reduction in ascorbic acid or vitamin C levels during *Plasmodium* infection is one of the proposed mechanisms that relate to the reduction in vitamin E in patients with malaria. Ascorbic acid or vitamin C is maintained in its natural form with ascorbate; therefore, ascorbic acid deficiency may hinder the regeneration of vitamin E and decrease membrane function [50]. A negative relationship between serum vitamin E and the reactive lipid metabolite malondialdehyde has been reported, indicating that reduced vitamin E augmented lipid peroxidation and contributed to erythrocyte destruction [43]. This evidence supported that vitamin E may act as an antioxidant, protecting the cell from oxidative stress caused by *Plasmodium* infection.

Although most of the included studies showed decreased vitamin E in malaria patients compared with those who were not infected, few studies reported the opposite results [32,45]. Between these two studies, one study reported higher plasma levels of vitamin E in malaria patients than in those who were not infected. The authors suggested that little vitamin E was consumed during *Plasmodium* infection [32]. Another study also reported higher levels of plasma vitamin E in children with *P. falciparum* malaria, but the levels of vitamin E in erythrocyte membranes were lower than in uninfected controls [45]. The authors proposed that higher plasma levels of vitamin E during *Plasmodium* infections might be due to the release of membrane vitamin E caused by acute hemolysis of the erythrocytes [45].

Interestingly, the subgroup meta-analysis of participants enrolled in the included studies showed comparable levels of vitamin E between pregnant women who were infected and those who were not infected with malaria. This result was suggested to be explained by the fact that pregnant women showed a hyperlipidemic state during pregnancy, and vitamin E was also increased in this state as it is a fat-soluble vitamin [51]. In addition, it is probable that the enhancement of vitamin E dominates its use as an antioxidant defense molecule [46]. Another explanation was that the majority of pregnant women had asymptomatic infections, which might result in them exhibiting lower oxidative stress levels than those with symptomatic infections. Hence, a small amount of vitamin E may be diminished or non-significantly lower in pregnant women. 

There were inconsistencies regarding the difference in vitamin E levels among patients suffering from either *P. falciparum* or *P. vivax* malaria. Three studies reported vitamin E levels among patients suffering from either *P. falciparum* or *P. vivax* malaria [37,41,43]. One study showed that vitamin E levels were significantly decreased in *P. falciparum* malaria as compared with *P. vivax* malaria [37]. Similarly, the other two studies showed lower mean vitamin E levels in *P. falciparum* malaria as compared with *P. vivax* malaria [41,43]. It has been suggested that *P. falciparum* infection led to more oxidative stress than *P. vivax* infection [43]. A previous study showed higher levels of lipid peroxides in patients with *P. falciparum* infections as compared with those with *P. vivax* infections. Therefore, a higher amount of vitamin E may be used to counteract high oxidative levels, which lower the levels of blood vitamin E. Additional investigations are required to confirm the difference in vitamin E levels between *P. falciparum* and *P. vivax* infections. Furthermore, due to the increased oxidative stress and reduction in antioxidant vitamin E during *Plasmodium* infection, antioxidant vitamin E supplementation or foods rich in vitamin E are being recommended for the management of patients with malaria.

There was little information regarding the association between vitamin E levels and parasite density. Three studies reported that vitamin E levels increased significantly as malaria density increased [39,40,48]. This association suggested that patients with an increased number of parasites might be more susceptible to higher levels of oxidative stress. Nevertheless, the positive association between vitamin E levels and parasite density raised the question about the role of vitamin E in different parasite densities as the meta-analysis results showed decreasing vitamin E levels in participants who were infected with malaria. As the information on the association was limited by those three studies, future studies with a higher number of malaria cases are needed to confirm the direction of this relationship. There was also little information regarding the association between vitamin E levels and severity levels of malaria. Based on the two studies that reported vitamin E levels among patients with severe malaria in comparison with those experiencing less severe forms of the disease [32,44], there was inconsistency regarding the vitamin E levels among various levels of malaria severity. Despite the meta-analysis indicating a considerable reduction in blood levels of vitamin E in patients with severe malaria in comparison with those with less severe disease, the results of an individual study showed lower vitamin E levels in severe malaria as compared with non-severe malaria [32], but a significant difference was not found by another study [44]. The inconsistency in the results between studies might reflect the differences in nutritional status and diet between the different studies’ locations [44].

This systematic review and meta-analysis included limitations. First, the meta-analysis’s heterogeneity remained in the results of the present study. Second, the publication bias in the meta-analysis might affect the conclusions made in this study. A small number of studies that examined the relationship between vitamin E among patients with varying levels of clinical severity and different species of *Plasmodium* causing malaria were incorporated in the analysis. Hence, more studies are needed to investigate the confidence of the meta-analysis findings.

## 5. Conclusions

The findings of the systematic review and meta-analysis strongly suggest that vitamin E levels are significantly reduced in malaria patients, with a more pronounced decrease observed in cases of severe malaria. However, the type of malaria parasite, specifically *P. falciparum* or *P. vivax*, did not appear to influence the levels of vitamin E. The present study highlights the significant reduction in vitamin E levels that may be related to the pathogenesis of malaria and suggests that improved vitamin E status might be beneficial for improving disease outcomes.

## Figures and Tables

**Table 1:** Overview of the characteristics of the studies.

Characteristics	n. (23)	%
**Year of publication**		
2011–2022	9	39.1
2000–2010	7	30.4
Before 2000	7	30.4
**Study designs**		
Case-control studies	11	47.8
Cross-sectional studies	10	43.5
Cohort studies	2	8.70
**Study areas**		
**Africa**	**13**	**56.5**
Nigeria	12	92.3
Kenya	1	7.70
**Asia**	**8**	**34.8**
India	5	62.5
Thailand	2	25.0
Vietnam	1	12.5
Europe	**2**	**8.70**
***Plasmodium* spp.**		
*P. falciparum*	16	69.6
*P. falciparum, P. vivax*	4	17.4
*P. falciparum, P. vivax*, *P. ovale*	1	4.34
Not specified	2	8.70
**Participants**		
Children	9	39.1
Adults	7	30.4
Pregnant women	4	17.4
All age groups	1	4.34
Pregnant and nonpregnant women	1	4.34
Not specified	1	4.34
**Clinical status**		
Symptomatic malaria	16	69.6
Asymptomatic malaria	1	4.34
Status not defined	6	26.1
**Methods for malaria detection**		
Microscopy	22	95.7
Not specified	1	4.30
**Form of vitamin E**		
α-tocopherol	9	39.1
Total tocopherols	2	8.70
Form was not specified	12	52.2

**Table 2:** Subgroup analyses of vitamin E levels between malaria cases and uninfected controls.

Subgroup Analyses	*p*-Value	Cohen’s d (95% CI)	I^2^ (%)	Number of Studies
Continent				
Africa	<0.01	−3.10, −4.54–(−1.66)	98.95	12
Asia	<0.01	−1.74, −2.95–(−0.52)	97.81	8
Age group				
Children	<0.01	−3.97, −5.35–(−2.59)	98.76	9
Adults	<0.01	−3.66, −5.07–(−2.25)	94.41	6
Pregnant women	0.43	0.88, −1.31–3.07	98.92	4
Children and adults	N/A	−0.89, −1.39–(−0.40)	N/A	1
Not specified	N/A	−1.85, −2.48–(−1.22)	N/A	1
*Plasmodium* species				
*P. falciparum*	<0.01	−2.79, −3.99–(−1.60)	98.81	14
*P. falciparum, P. vivax, P. Ovale*	N/A	−8.02, −9.57–(−6.47)	N/A	1
*P. falciparum, P. vivax*	<0.01	−2.64, −4.32–(−0.95)	96.88	4
Not specified	0.93	−0.16, −3.52–3.20	98.75	2
Forms of vitamin E				
Total tocopherols	0.30	−13.66, −39.24–11.92	99.68	2
α-tocopherol	0.08	−0.69, −1.48–(−0.09)	96.20	8
Not specified	<0.01	−2.87, −4.81–(−0.93)	99.01	11

Abbreviations: CI, confidence interval; N/A, not assessed.

## Data Availability

All study-related data can be found within the manuscript and its supplementary files.

## Supplementary Materials

The following supporting information can be downloaded at https://www.mdpi.com/article/10.3390/nu15153472/s1: Table S1: Search terms; Table S2: Details of the included studies; Table S3: Quality of included studies; Table S4: Meta-regression results; Figure S1: Malaria vs. uninfected controls_confunnel.
