# Supplementary material for: Effect of Malaria on Blood Levels of Vitamin E: A Systematic Review and Meta-Analysis
Source: Nutrients. 2023 Aug 5;15(15):3472. doi: 10.3390/nu15153472 (PMC10421180; doi:10.3390/nu15153472)
Supplement: Supplementary file 1 [file nutrients-15-03472-s001.zip › Table S1. Search terms.docx]

**Effect of malaria on blood levels of vitamin E: A systematic review and meta-analysis**

**Running title:** Vitamin E and malaria

Manas Kotepui^1^, Frederick Ramirez Masangkay^2^, Aongart Mahittikorn^3^*, Kwuntida Uthaisar Kotepui^1^*

^1^Medical Technology, School of Allied Health Sciences, Walailak University, Tha Sala, Nakhon Si Thammarat, Thailand

^2^Department of Medical Technology, Faculty of Pharmacy, Santo Tomas, Manila, Philippines

^3^Department of Protozoology, Faculty of Tropical Medicine, Mahidol University, Bangkok, Thailand

***Corresponding author**

Manas Kotepui: manas.ko@wu.ac.th

Frederick Ramirez Masangkay: [frederick_masangkay2002@yahoo.com](mailto:frederick_masangkay2002@yahoo.com)

Aongart Mahittikorn: [aongart.mah@mahidol.ac.th](mailto:aongart.mah@mahidol.ac.th)

Kwuntida Uthaisar Kotepui: [kwuntida.ut@wu.ac.th](mailto:kwuntida.ut@wu.ac.th)

**Table S1. Search strategy**

**General search terms**

(“Vitamin E” OR Tocopherols OR alpha-Tocopherol OR beta-Tocopherol OR gamma-Tocopherol OR Tocotrienols) AND (malaria OR Plasmodium OR "Remittent Fever" OR "Marsh Fever" OR Paludism)

**PubMed**

**31 March 2023**

| **No.** | **Query** | **Results** |
| --- | --- | --- |
| 3 | #1 AND #2 | 95 |
| 2 | (((malaria) OR (malaria[MeSH Terms])) OR (Plasmodium)) OR (Plasmodium[MeSH Terms]) | 121,511 |
| 1 | (Vitamin E) OR (Vitamin E[MeSH Terms]) | 47,021 |

**Scopus**

**31 March 2023**

| **No.** | **Query** | **Results** |
| --- | --- | --- |
| 3 | 1 AND 2 | 283 |
| 2 | TITLE-ABS-KEY (malaria OR plasmodium OR "remittent fever" OR "marsh fever" OR paludism) | 155,404 |
| 1 | TITLE-ABS-KEY ("vitamin e" OR tocopherols OR alpha-tocopherol OR beta-tocopherol OR gamma-tocopherol OR tocotrienols) | 110,144 |

**MEDLINE**

| **No.** | **Search terms/Search strategy** | **Date** |
| --- | --- | --- |
| 1 | (“Vitamin E” OR Tocopherols OR alpha-Tocopherol OR beta-Tocopherol OR gamma-Tocopherol OR Tocotrienols) AND (malaria OR Plasmodium OR "Remittent Fever" OR "Marsh Fever" OR Paludism)  Search results: 116 | **31 March 2023** |

**Embase**

**31 March 2023**

| **No.** | **Query** | **Results** |
| --- | --- | --- |
| 3 | #1 AND #2 | 288 |
| 2 | malaria OR plasmodium OR 'remittent fever' OR 'marsh fever' OR paludism | 161,766 |
| 1 | 'vitamin e'/exp OR 'vitamin e' OR 'tocopherols'/exp OR tocopherols OR 'alpha tocopherol'/exp OR 'alpha tocopherol' OR 'beta tocopherol'/exp OR 'beta tocopherol' OR 'gamma tocopherol'/exp OR 'gamma tocopherol' OR 'tocotrienols'/exp OR tocotrienols | 94,504 |

**Ovid**

| **No.** | **Search terms/Search strategy** | **Date** |
| --- | --- | --- |
| 1 | (“Vitamin E” OR Tocopherols OR alpha-Tocopherol OR beta-Tocopherol OR gamma-Tocopherol OR Tocotrienols) AND (malaria OR Plasmodium OR "Remittent Fever" OR "Marsh Fever" OR Paludism) {No Related Terms}  limit to (articles with abstracts and original articles)  Search results: 338 | **31 March 2023** |
