# Supplementary material for: Effect of Malaria on Blood Levels of Vitamin E: A Systematic Review and Meta-Analysis
Source: Nutrients. 2023 Aug 5;15(15):3472. doi: 10.3390/nu15153472 (PMC10421180; doi:10.3390/nu15153472)
Supplement: Supplementary file 1 [file nutrients-15-03472-s001.zip › Table S4. Meta-regression results.docx]

**Effect of malaria on blood levels of vitamin E: A systematic review and meta-analysis**

**Running title:** Vitamin E and malaria

Manas Kotepui^1^, Frederick Ramirez Masangkay^2^, Aongart Mahittikorn^3^*, Kwuntida Uthaisar Kotepui^1^*

^1^Medical Technology, School of Allied Health Sciences, Walailak University, Tha Sala, Nakhon Si Thammarat, Thailand

^2^Department of Medical Technology, Faculty of Pharmacy, Santo Tomas, Manila, Philippines

^3^Department of Protozoology, Faculty of Tropical Medicine, Mahidol University, Bangkok, Thailand

Manas Kotepui: manas.ko@wu.ac.th

Frederick Ramirez Masangkay: [frederick_masangkay2002@yahoo.com](mailto:frederick_masangkay2002@yahoo.com)

Aongart Mahittikorn: [aongart.mah@mahidol.ac.th](mailto:aongart.mah@mahidol.ac.th)

Kwuntida Uthaisar Kotepui: [kwuntida.ut@wu.ac.th](mailto:kwuntida.ut@wu.ac.th)

**Table S4. Meta-regression results**

| **Meta-analysis of vitamin E** | **Covariates** | **P value** | **R-squared (%)** | **tau2** | **I^2^ (%)** |
| --- | --- | --- | --- | --- | --- |
| **Malaria vs uninfected controls** | Publication years | 0.33 | 0 | 5.40 | 98.74 |
|  | Study design | 0.76 | 0 | 5.66 | 98.74 |
|  | Continent | 0.03 | 1.94 | 4.96 | 98.69 |
|  | Participants ‘group | <0.01 | 16.69 | 4.22 | 98.42 |
|  | *Plasmodium* spp. | 0.05 | 5.58 | 4.78 | 98.66 |
|  | Clinical status | 0.71 | 0 | 5.29 | 98.72 |
|  | Method for *Plasmodium* spp. | 0.39 | 6.68 | 4.72 | 98.61 |
|  | Form of vitamin E | <0.01 | 6.24 | 6.24 | 98.81 |
