# Supplementary figures and images for: Effect of Malaria on Blood Levels of Vitamin E: A Systematic Review and Meta-Analysis
Source: Nutrients. 2023 Aug 5;15(15):3472. doi: 10.3390/nu15153472 (PMC10421180; doi:10.3390/nu15153472)

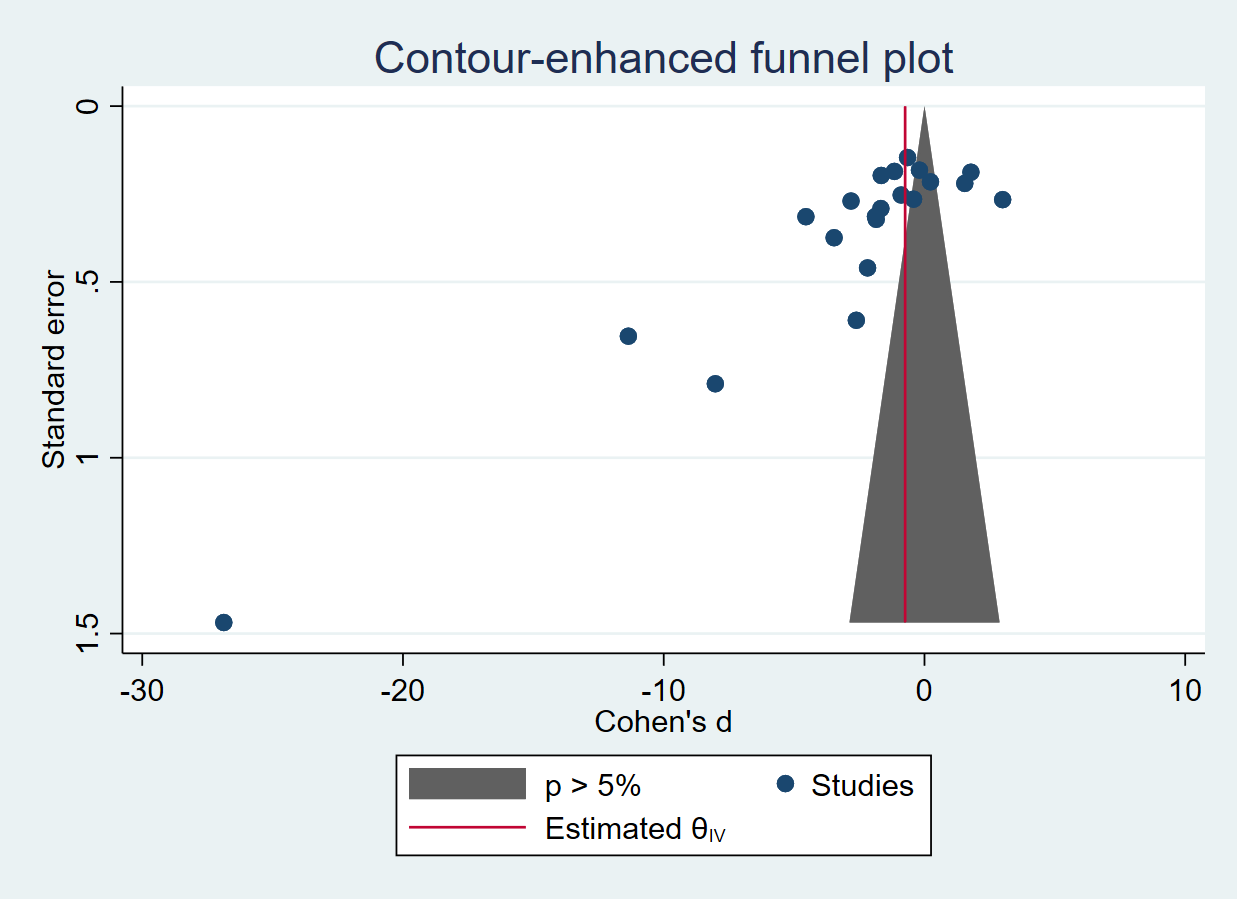

Supplement: Supplementary file 1 [file nutrients-15-03472-s001.zip › nutrients-2490768-Supplementary Figure S1. Malaria vs. uninfected controls_confunnel.tif]
